# Supplementary material for: The Systems Biology Research Tool: evolvable open-source software
Source: BMC Syst Biol. 2008 Jun 29;2:55. doi: 10.1186/1752-0509-2-55 (PMC2446383; doi:10.1186/1752-0509-2-55)
Supplement: Additional file 1 — SBRT Archive. An archive of the current version of the Systems Biology Research Tool. [file 1752-0509-2-55-S1.zip › sbrt-1.4.0/doc/developers_guide/api/sbrt/kernel/util/AbstractProgressMonitor.html]

AbstractProgressMonitor


|  |  |  |
| --- | --- | --- |
| **Overview** \| **Package** \| **Class** \| **Use** \| **Tree** \| **Deprecated** \| **Index** \| **Help** | |  |
| PREV CLASS   **NEXT CLASS** | **FRAMES**    **NO FRAMES**     **All Classes** |  |
| SUMMARY: NESTED \| FIELD \| CONSTR \| METHOD | DETAIL: FIELD \| CONSTR \| METHOD |  |


---


## sbrt.kernel.util Class AbstractProgressMonitor

```
java.lang.Object
  sbrt.kernel.util.AbstractProgressMonitor
```

**All Implemented Interfaces:**: MutableProgressMonitor, ProgressMonitor

**Direct Known Subclasses:**: DiscreteProgressMonitor, PercentProgressMonitor

---

``` public abstract class AbstractProgressMonitor extends java.lang.Object implements MutableProgressMonitor ```

This abstract class is a skeleton implementation of `ProgressMonitor`.
This class provided methods for counting events and measuring elapsed times.

**Author:**
:   This class was written and documented by
    Jeremiah Wright while in the Wagner lab.

---

**Constructor Summary**

`AbstractProgressMonitor()`
  
          Constructs a new progress monitor.


| **Method Summary** | |
| --- | --- |
| `int` | `getCount()`             Returns the number of times of `increment()` has been called. |
| `long` | `getDuration()`             Returns the elapsed time between the calls of `start()` and `stop()`. |
| `void` | `increment()`             Causes this progress monitor to increment an internal counter. |
| `void` | `resetCount()`             Causes this progress monitor to reset it's internal counter to 0. |
| `void` | `start()`             Causes this progress monitor to start monitoring a duration of time. |
| `boolean` | `started()`             Indicates if this progress monitor has already been started. |
| `void` | `stop()`             Causes this progress monitor to stop monitoring a duration of time. |
| `boolean` | `stopped()`             Indicates if this progress monitor has already been stopped. |

**Methods inherited from class java.lang.Object**

`clone, equals, finalize, getClass, hashCode, notify, notifyAll, toString, wait, wait, wait`

**Methods inherited from interface sbrt.kernel.util.MutableProgressMonitor**

`asUnmodifiable`

**Methods inherited from interface sbrt.kernel.util.ProgressMonitor**

`getProgress`

**Constructor Detail**

### AbstractProgressMonitor

```
public AbstractProgressMonitor()
```

:   Constructs a new progress monitor.


**Method Detail**

### getCount

```
public int getCount()
```

:   Returns the number of times of `increment()` has been called.

    :   **Returns:**: the number of times of `increment()` has been called.

---


### increment

```
public void increment()
```

:   Causes this progress monitor to increment an internal counter.

    :   **Specified by:**: `increment` in interface `MutableProgressMonitor`
    :   **See Also:**: `getCount()`

---


### resetCount

```
public void resetCount()
```

:   Causes this progress monitor to reset it's internal counter to 0.

    :   **Specified by:**: `resetCount` in interface `MutableProgressMonitor`
    :   **See Also:**: `increment()`, `getCount()`

---


### start

```
public void start()
```

:   Causes this progress monitor to start monitoring a duration of time.

    :   **Specified by:**: `start` in interface `MutableProgressMonitor`
    :   **Throws:**: `java.lang.IllegalStateException` - if `start()` was already called without a subsequent call to `stop()`.

---


### stop

```
public void stop()
```

:   Causes this progress monitor to stop monitoring a duration of time.

    :   **Specified by:**: `stop` in interface `MutableProgressMonitor`
    :   **Throws:**: `java.lang.IllegalStateException` - if `start()` was never called, or if `stop()` has already been called since the last call to `start()`.

---


### started

```
public boolean started()
```

:   Indicates if this progress monitor has already been started.

    :   **Specified by:**: `started` in interface `ProgressMonitor`
    :   **Returns:**: `true` if this progress monitor has already been started; `false` otherwise.

---


### stopped

```
public boolean stopped()
```

:   Indicates if this progress monitor has already been stopped.

    :   **Specified by:**: `stopped` in interface `ProgressMonitor`
    :   **Returns:**: `true` if this progress monitor has already been stopped; `false` otherwise.

---


### getDuration

```
public long getDuration()
```

:   Returns the elapsed time between the calls of `start()` and
    `stop()`. If `start()` was never called, `0` is
    returned.
    If `stop()` hasn't been called since the last call to
    `start()`, the elapsed time since the last call to `start()`
    is returned.

    :   **Specified by:**: `getDuration` in interface `ProgressMonitor`
    :   **Returns:**: the elapsed time between the calls of `start()` and `stop()`.


---


|  |  |  |
| --- | --- | --- |
| **Overview** \| **Package** \| **Class** \| **Use** \| **Tree** \| **Deprecated** \| **Index** \| **Help** | |  |
| PREV CLASS   **NEXT CLASS** | **FRAMES**    **NO FRAMES**     **All Classes** |  |
| SUMMARY: NESTED \| FIELD \| CONSTR \| METHOD | DETAIL: FIELD \| CONSTR \| METHOD |  |


---
